# Preparation and Characterization of Nanoparticle-Doped Polymer Inclusion Membranes. Application to the Removal of Arsenate and Phosphate from Waters

**DOI:** 10.3390/ma14040878

**Published:** 2021-02-12

**Authors:** Enriqueta Anticó, Ruben Vera, Francisco Vázquez, Clàudia Fontàs, Changyong Lu, Josep Ros

**Affiliations:** 1 Departament de Química, Universitat de Girona, Campus Montilivi, 17003 Girona, Spain; ruben.vech@gmail.com (R.V.); francisco5027@gmail.com (F.V.); claudia.fontas@udg.edu (C.F.); 2 Department of Plants and Environmental Sciences, University of Copenhagen, DK-1871 Frederiksberg, Denmark; clu@plen.ku.dk; 3 Departament de Química, Universitat Autònoma de Barcelona (UAB), 08193 Cerdanyola del Vallès, Spain; josep.ros@uab.cat

**Keywords:** polymer inclusion membranes, nanoparticles, multiwalled carbon nanotubes, arsenate, phosphate, removal

## Abstract

Nanoparticle-doped polymer inclusion membranes (NP-PIMs) have been prepared and characterized as new materials for the removal of arsenate and phosphate from waters. PIMs are made of a polymer, cellulose triacetate (CTA), and an extractant, which interacts with the compound of interest. We have used the ionic liquid (IL) trioctylmethylammonium chloride (Aliquat 336) as the extractant and have investigated how the addition of nanoparticles can modify membrane properties. To this end, inorganic nanoparticles, such as ferrite (Fe_3_O_4_), SiO_2_ and TiO_2_, and multiwalled carbon nanotubes (MWCNTs), were blended with the polymer/extractant mixture. Scanning electron microscopy (SEM), infrared spectroscopy (FT-IR), and contact angle measurements have been used to characterize the material. Moreover, PIM stability was checked by measuring the mass loss during the experiments. Since Aliquat 336 acts as an anion exchanger, the NP-PIMs have been explored in two different applications: (i) as sorbent materials for the extraction of arsenate and phosphate anions; (ii) as an organic phase for the separation of arsenate and phosphate in a three-phase system. The presence of oleate-coated ferrite NP in the PIM formulation represents an improvement in the efficiency of NP-PIMs used as sorbents; nevertheless, a decrease in the transport efficiency for arsenate but not for phosphate was obtained. The ease with which the NP-PIMs are prepared suggests good potential for future applications in the treatment of polluted water. Future work will address three main aspects: firstly, the implementation of the Fe_3_O_4_-PIMs for the removal of As(V) in real water containing complex matrices; secondly, the study of phosphate recovery with other cell designs that allow large volumes of contaminated water to be treated; and thirdly, the investigation of the role of MWCNTs in PIM stability.

## 1. Introduction

Polymer inclusion membranes (PIMs) have been employed for the extraction of a wide variety of compounds from anions or metallic species to small organic molecules [1]. These membranes are made of a polymer, normally polyvinyl chloride (PVC) or cellulose triacetate (CTA), which provides mechanical strength, an extractant, which is entrapped within the polymeric matrix and is responsible for binding the species of interest by complexation or ion-pair formation, and sometimes a plasticizer or modifier [2]. Nowadays, there is increasing interest in PIMs thanks to their easy preparation, low cost, versatility, improved stability compared to conventional liquid membranes, and high efficiency. PIMs are used for the separation of two aqueous phases: the sample or source solution, containing the analyte, and a receiving solution where the analyte is back-extracted. As long as a chemical potential difference exists on both sides of the membrane, the analyte can be transported against its concentration gradient (up-hill transport or chemical pumping). The extractant used in the PIM has a key role in facilitating selective transport. For anions, PIMs commonly include an anion exchanger as the extractant, and the driving force for the transport is a chemical gradient of a negatively charged species added to the receiving phase at a high concentration [3]. 

PIMs have also been used as sorbents; in this case, the PIM is contacted with the aqueous solution first, and the recovery of the extracted species can take place in a separate stage (not simultaneously). The target species is separated from the aqueous phase and retained in the polymeric matrix, thanks to the presence of the extractant that selectively binds the ion. This approach, called solid phase extraction, has been applied to Zn and Cr(VI) extraction with PIMs embedded with di-(2-ethylhexyl)phosphoric acid (D2EHPA) [4] and with trioctylmethylammonium chloride (Aliquat 336) [5], respectively. In the same manner, Cd [6], Hg [7], and Cr [8] preconcentration with PIMs containing different extractants has also been demonstrated, and these last three studies were aimed at monitoring metals by measuring analytical signals related to metal concentrations directly in the PIM phase, which represents a great advantage for monitoring purposes since no elution step is needed.

Arsenate and phosphate are soluble species, considered as pollutants, which are present in waters from both natural and anthropogenic sources. The abundance of arsenic in natural soils is usually below 10 mg·Kg^−1^ [9]. Although it is relatively scarce, it is found in high concentrations in sulfide deposits, where it is present as the native element or as an alloy, or in the form of arsenic oxides, arsenic sulfides, and metal arsenides [10]. Phosphorus, on the other hand, has an abundance of 0.10–0.12% (on a weight basis), existing as inorganic phosphate minerals (phosphorite and apatite) and phosphorus containing organic compounds [11].

In aqueous solution, arsenic forms the oxo-anions, arsenite and arsenate. From the pKa values, and taking into account the pH of natural waters, the neutral species of As3+, and the species H_2_ASO_4_^−^ and HAsO_4_^2−^ are expected to predominate in the cases of As3+ and As5+, respectively [10]. According to the redox potential, arsenite should be stable at moderate reducing conditions whereas arsenate is stable in oxidized aqueous media such as surface waters. It is important to point out that arsenate has a strong affinity for oxides and oxyhydroxydes, probably as a result of the formation of inner-sphere mononuclear or binuclear monodentate-bidentate complexes [12]. Due to its natural occurrence and its use in agriculture (as a herbicide, insecticide, and wood preservative) and industry, dangerously high levels of arsenic are found in the ground water of several different countries, including Bangladesh, India, China, and Argentina. Arsenic has been classified as carcinogenic and regulated by the WHO in drinking water at a maximum level of 10 µg L^−1^. Long term exposure to arsenic contaminated drinking water causes pigmentation changes, skin thickening, neurological disorders, muscular weakness, loss of appetite, and nausea [13]. 

The chemistry in solution of phosphate is similar to arsenate (acid-base equilibria), and under oxidized conditions dissolved phosphate will be present as an aqueous species of similar charge and chemical structure over the pH range of natural waters as arsenate [10]. P (together with N) is essential in photosynthetic processes, cell growth, metabolism, and protein synthesis [14]. Phosphate is mainly found in surface waters due to natural or anthropogenic sources. The primary source of P today is from the massive inputs of fertilizers onto agricultural land. P in soil may be lost by leaching, runoff, and erosion, and with entry into aquatic systems, P triggers eutrophication before being cycled or buried as sediments [15]. A recent report [16] indicates that P levels is one of the quality elements leading to the poor ecological status of a large proportion of rivers in Europe. Despite the negative effect of P on the environment, the recovery of phosphate from wastewaters can be viewed as a possibility to take advantage of P-rich wastes [17].

Technologies for water treatment in the case of the removal of arsenate and phosphate rely basically on adsorption onto solid sorbents [18,19,20,21]. Among the sorbent materials, metal hydroxyoxides are used to taking advantage of their affinity towards the target anions. However, the material reuse is not always accomplished, generating a high amount of waste and making the process less cost-effective. Moreover, the recovery and valorization of the species (in the case of phosphate) is only possible if an adequate elution is feasible [22]. The use of membrane separation systems is advantageous since the species of interest is recovered in an aqueous receiving phase. Polymeric membranes blended with inorganic materials, such as molecular sieves, zeolite, metal oxides, silica nanoparticle, carbon nanotubes, among others, have been described for gas separation [23,24], showing properties that are superior to the polymeric membranes on their own. Moreover, the modification of PIMs with nanomaterials (from now on called NP-PIMs) has been performed for purposes other than gas separation. The antibacterial and fouling resistance poly-(polyvinylidene fluoride) (PVDF) composite membranes doped with silver and multi-walled carbon nanotubes have been investigated [25]. Silver nanocomposite polymer inclusion membranes containing D2EHPA were recently evaluated for the transport of cationic species. The authors investigated the influence of silver nanoparticle loadings on the PIMs appearance, flexibility, roughness, and hydrophilicity [26]. The presence of Mg-Al-CO_3_ layered double hydroxide incorporated in a PIM composed of PVC and Aliquat 336 was prepared to promote the extraction efficiency towards cyanide [27]. PIMs doped with reduced graphene oxide and modified graphene quantum dots were prepared and used for the recovery of Cr(VI) [28,29,30] using calix[4]arenes as extractants. The catalytic activity of membranes prepared with Ag and Pd nanoparticles has also been exploited [31]. A method for coating a polymer inclusion membrane with palladium nanoparticles has also been described [32]. As for gold nanoparticles, the in situ formation of gold nanoparticles in polymer inclusion membranes has been presented (Specht), and the method has been exploited in a label-free potentiometric immunosensor for Salmonella typhimurium detection [33]. So far, to the best of our knowledge, no other NP-PIMs have been described that have specifically been designed for the removal of anions of environmental interest. The application of PIMs with improved stability and selectivity would be an alternative to other membrane technologies used for water cleaning purposes. The preparation of NP-PIMs, their characterization, and examination of the performance at laboratory level is a first important stage for future uses.

In the present study, we investigate the effect of the addition of different inorganic NPs such as ferrite nanoparticles (Fe_3_O_4_), commercially available TiO_2_ and SiO_2_ NPs, and also carbon nanotubes (MWCNTs), in PIMs based on the polymer CTA and the anion exchanger Aliquat 336 for phosphate and arsenate removal. Aliquat 336 has been chosen as the extractant due to its good performance in other works dealing with anion transport [5,34]. The NP-PIMs have been characterized by SEM, FTIR, and contact angle measurements. The stability of the NP-PIMs and their applications in the removal of arsenate and phosphate from water samples will be demonstrated in both adsorption and transport experiments.

## 2. Materials and Methods

### 2.1. Reagents and Solution

The reagents used for the preparation of the simulated natural water (SNW) were sodium sulfate (Merck, Madrid, Spain), calcium chloride hexahydrate (Panreac, Barcelona, Spain), and sodium hydrogen carbonate (Merck, Madrid, Spain). The concentration of the salts in SNW was: 2 × 10^−3^ M NaHCO_3,_ 0.75 × 10^−3^ M CaCl_2_, and 0.25 × 10^−3^ M Na_2_SO_4_, and the measured pH was 8.3.

For the preparation of PIMs, the following reagents were used: cellulose triacetate (Acros Organics, Fisher Scientific, Madrid, Spain), and the extractant trioctylmethylammonium chloride (Aliquat 336^®^) (Sigma-Aldrich, Steinheim, Germany). Chloroform (Panreac, Bercelona, Spain) was used as a solvent and ultrapure water with resistivity ≥18 μS cm^−1^ was taken from a MilliQ system (Millipore Ibérica S.A., Barcelona, Spain).

Standard solutions of 1000 mg L^−1^ of As and P (Sigma-Aldrich, Steinheim, Germany) were used for the measurement of the elements in the aqueous solutions.

All the reagents were of analytical grade purity.

### 2.2. Nanoparticles

We used different types of nanoparticles for the modifications of PIMs. SiO_2_ (99.5% purity) and TiO_2_ (99.5% purity), bearing particle size 10–20 nm and <100 nm, respectively, were purchased from Sigma-Aldrich (Steinheim, Germany). Multiwalled carbon nanotubes (MWCNTs) (6–9 μm diameter and 5 μm length) were also from Sigma-Aldrich (Steinheim, Germany).

Oleate-coated ferrite NPs were synthesized in the laboratory of Prof. Ros (UAB, Spain) [35]. Oleic acid (3 × 10^−3^ mol), oleylamine (3 × 10^−3^ mol), and triethyleneglycol (5 × 10^−3^ mol) were added to a 20 mL of a solution of iron(III)acethylacetonate (1 × 10^−3^ mol) in benzylether. The final mixture was transferred to a magnetically stirred two-neck round-bottom flask equipped with a water-cooled condenser and purged with N_2_ for 30 min and heated under N_2_ to 200 °C (with a heating rate of 1 °C min^−1^) for 30 min. After this time, the mixture was refluxed at 265 °C with the same heating rate under N_2_ for another 30 min. The black solution was cooled down to room temperature and ethanol was added to precipitate solid Fe_3_O_4_ nanoparticles. The mixture was centrifuged (1000 rpm, 10 min), the solid was filtered off and dispersed in 20 mL of hexane containing 0.14 mmol of oleic acid and 0.152 mmol of oleylamine. The dispersion obtained was centrifuged to 6000 rpm for 10 min and the insoluble part was filtered off. Monodispersed Fe_3_O_4_ nanoparticles were precipitated adding an excess of ethanol to the solution, collected with centrifugation (1000 rpm, 10 min), and dispersed in 20 mL of hexane.

The characterization of the newly synthesized NP was performed by X-ray powder diffraction (XRD) (D5000 Siemens X-ray diffractometer, Siemens AG, Munich, Germany), dynamic light scattering (DLS) (Zeta-sizer Nano Z system, Malvern Instruments, Malvern, UK), transmission electron microscopy (TEM) (JEOL 1210 TEM, Tokyo, Japan), Fourier transform infrared spectroscopy (FTIR) (Bruker Tensor27 Fourier transform infrared spectrometer, Bruker Physik AG, Karlsruhe, Germany), and thermogravimetry techniques (NETZSCH-STA 449 F1 Jupiter thermal analysis system, Burlington, MA, USA) [35]. The amount of organic ligand on the surface of the Fe_3_O_4_ nanoparticles was determined by thermogravimetric analysis. The organic compound started to decompose at 200 °C and fully oxidized at 400 °C, leading to a dramatic weight loss of 19.17%. Accordingly, the amount of Fe_3_O_4_ nanoparticles is 80.83%, giving a final concentration of Fe in the NP hexane solution of 20 mM. The absorption peaks observed in the FT-IR curve indicate that the oleic acid is absorbed on the Fe_3_O_4_ nanoparticles, which results in a good dispersion of Fe_3_O_4_ in hexane [35]. The average diameter of the obtained nanoparticles is 8.2 nm as measured by DLS.

**Figure 1:**
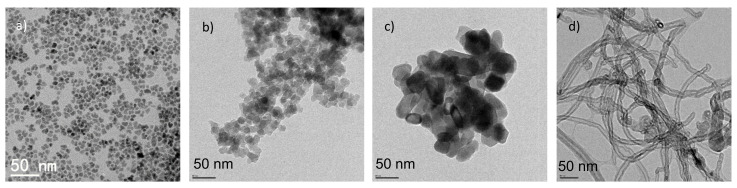
TEM image of: (**a**) synthesized Fe_3_O_4_ nanoparticle; (**b**) SiO_2_, NP in ethanol; (**c**) TiO_2_ NP in ethanol; and (**d**) multiwalled carbon nanotubes (MWCNTs) in ethanol.

Figure 1 shows the TEM image of dispersed Fe_3_O_4_ nanoparticles in hexane and of commercial SiO_2_, TiO_2_, and MWCNTs dispersed in ethanol.

### 2.3. NP-PIMs Preparation

**Figure 2:**
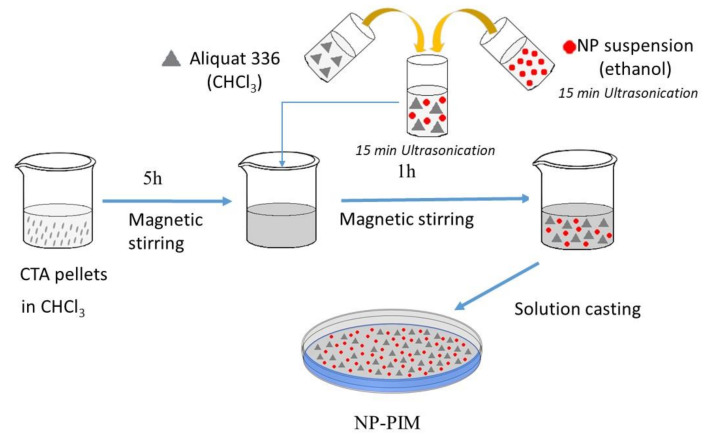
Scheme of the preparation of nanoparticle-doped polymer inclusion membranes (NP-PIMs).

PIMs were prepared by dissolving 155 mg of CTA in chloroform (5 h) and 0.7 mL of 0.5 M solution of Aliquat 336 in CHCl_3_ (141 mg), which was previously blended with the corresponding amount of nanoparticles in suspension (see Figure 2). The suspension was prepared by using the appropriate amount of NP (see Table 1) in 5 mL ethanol (Panreac, Barcelona, Spain). In the case of Fe_3_O_4_ NP, a volume of the solution in hexane (Panreac, Barcelona, Spain) was employed. The suspension was homogenized in an ultrasonic bath for at least 15 min, then Aliquat 336 solution was added, homogenized, and finally added to the polymer solution. The mixture was poured into a 7.0 cm diameter flat bottom glass Petri dish, which was set horizontally and covered loosely. The solvent was allowed to evaporate over 24 h at room temperature, and the resulting film was then carefully peeled off the bottom and taken for further studies.

The composition of the PIMs prepared is presented in Table 1.

### 2.4. NP-PIMs Characterization

SEM images were obtained with a field emission scanning electron microscope (Hitachi, S-4100, Tokyo, Japan). Samples were placed on a stub and coated with carbon (model K950 turbo evaporator, Emitech, Lohmar, Germany). Digital images were collected and processed by the Quartz PCI 5.1 software

Changes in the hydrophobic character of the membrane surfaces associated to both the composition of IL and the NP modification were determined from contact angle measurements, which were performed by the tensile drop method using distilled water drops of 5 µL and a DSSA25 drop-shape analyzer (Krüss GmbH, Hamburg, Germany) equipped with a video system. The value given is the average of 120 measurements (60 s).

IR spectra were obtained with the aid of a diamond attenuated total reflectance accessory on an Agilent Cary 630 FTIR spectrometer (Agilent, Santa Clara, CA, USA). For each sample, 32 scans with a resolution of 8 cm^−1^ were recorded.

### 2.5. Procedures

#### 2.5.1. NP-PIM Stability Measurements

The stability of the different membranes under study was investigated by monitoring the mass change of PIMs containing the different IL. The PIM segments of an approximate area of 2 cm × 2 cm were taken for this purpose. Before the experiment, membrane pieces were carefully weighed. After 4 h contact with 15 mL of SNW, the membrane was removed from the solution and air-dried until constant weight was achieved.

Mass loss is calculated using Equation (1):

(1) $$Mass\;loss\;\left(\%\right)=\frac{W_{\left(0\right)}-W_{\left(t\right)}}{W_{\left(0\right)}}\times 100,$$

where *W_(o)_* is the initial membrane weight, and *W_(t)_* is the final membrane weight after each cycle.

#### 2.5.2. Sorption Experiments

The experiments were performed by contacting 15 mL of SNW containing separately 0.5 mg L^−1^ for As(V) and 1 mg L^−1^ for P, with the selected PIM (2 cm × 2 cm) in a glass tube under rotary agitation (40 rpm) for a predetermined time. After this, the remaining As or P was determined by inductively coupled plasma emission spectrometry using the ICP-OES 5100 spectrometer from Agilent (Santa Clara, CA, USA).

The percentage of extraction (*E%*) was calculated by using Equation (2):

(2) $$E\%=\;\frac{C_{feed,0}-\;C_{feed,t}}{C_{feed,0}}\;\times 100,$$

where *C_feed,0_* is the initial concentration in the solution, *C_feed,_*_t_ is the metal concentration in the solution after the time of contact.

#### 2.5.3. Transport Experiments

Transport was investigated using 50 mL of SNW spiked with 100 μg L^−1^ As(V) or 300 μg L^−1^ P(V) as a feed solution and 2.5 mL of 2 M NaCl as the receiving (or stripping) phase. A device previously described by Fontàs et al. was used [36]. The NP-PIM was placed at the bottom of the device contacting the feed phase while the receiving solution was placed inside the compartment. The transport and preconcentration of anionic species occurs through the NP-PIM from the feed phase to the receiving phase.

Transport efficiency (*TE*%) was determined by using Equation (3):

(3) $$TE\%=\;\frac{C_{strip,t}}{C_{feed,0}}\;\times \;\frac{V_{strip}}{V_{feed}}\;\times \;100,$$

where *C_strip,t_* denotes the As or P concentration in the receiving compartment at an elapsed time t and *C_feed,0_* is the initial As or P concentration in the feed phase, both measured by ICP-OES. *V_feed_* and *V_strip_* are the volumes of the solutions in the respective compartments.

### 2.6. Instruments and Apparatus

An inductively coupled plasma emission spectrometer (ICP-OES 5100, Agilent, Santa Clara, CA, USA) was used for the analysis of As and P concentrations in the aqueous solutions. The selected wavelengths were 193.696 nm for As and 213.618 nm for P.

A GLP-22 pH-meter (Crison, Barcelona, Spain) was used to measure the pH of the samples. A rotatory agitator (Dinko, Barcelona, Spain) was employed to perform the sorption experiments.

Transport experiments were performed using a magnetic multistirrer 15 from Fisher Scientific (Madrid, Spain).

All experiments were carried out at room temperature of 22 ± 1 °C.

## 3. Results and Discussion

### 3.1. NP-PIM Characterization

**Figure 3:**
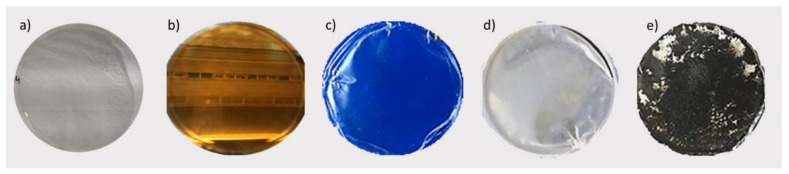
Images of the different PIMs: (**a**) M1; (**b**) M2 (0.5% Fe_3_O_4_); (**c**) M4 (1% SiO_2_); (**d**) M8 (1% TiO_2_); (**e**) M11 (MWCNTs).

PIMs prepared with CTA as the polymer and Aliquat 336 as the extractant are dense, with no apparent porosity, flexible, transparent, and with good mechanical properties as reported previously [37]. The presence of modifiers may change the flexibility and the visual aspect of the PIM, as observed in our study. The photographic images for M1 and the NP-PIMs are presented in Figure 3. The NP-PIMs appear transparent, brown in color when doped with Fe_3_O_4_ NP, and colorless for SiO_2_. When TiO_2_ was added, the NP-PIMs lost transparency and, partially, their flexibility. The same occurs for MWCNT NP-PIMs, but in this case, it appears black in color and the MWCNTs were not uniformly distributed.

**Figure 4:**
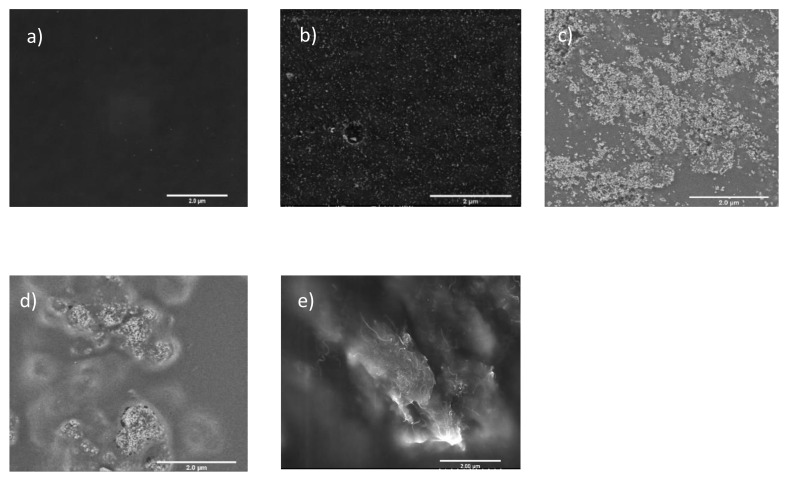
SEM images of the different PIMs: (**a**) M1; (**b**) M2 (0.5% Fe_3_O_4_); (**c**) M4 (1% SiO_2_); (**d**) M8 (1% TiO_2_); (**e**) M11 (MWCNTs).

The SEM images of the surface of the NP-PIMs are shown in Figure 4. The use of oleate-stabilized hexane solution of ferrite NP allowed the preparation of highly homogeneous PIMs with the NP particles well dispersed in the polymeric matrix. On the other hand, the formation of aggregates was observed for the other NP-PIMs.

To investigate the effect of the presence of the NP on the hydrophilic character of the membrane, contact angle measurements were carried out. The results are shown in Table 2. Values within the range of 15–25° were found, indicating the high hydrophilicity of the surface of the NP-PIMs. These values are in agreement with those found by Vera et al. and may be related to the high amount of Aliquat 336 present in the NP-PIM [37].

**Figure 5:**
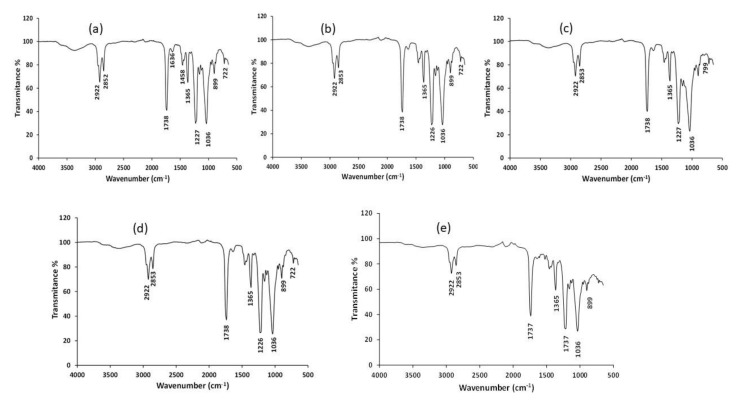
IR spectra of the PIMs: (**a**) M1; (**b**) M2 (0.5% Fe_3_O_4_); (**c**) M4 (1% SiO_2_); (**d**) M8 (1% TiO_2_); (**e**) M11 (MWCNTs).

IR spectroscopy was used to provide information on the bulk composition of the studied membranes. From FTIR spectra, the main bands could be observed, which correspond to those of the individual constituents of the membrane as reported previously [37]. The FTIR spectrum (from 4000 to 650 cm^−1^) of M1 and doped PIMs are shown in Figure 5. The stretching C-H vibrations are present in the 2960–2850 cm^−1^ region for all the membrane types. The band at 1227 cm^−1^, assigned to the quaternary ammonium group in Aliquat 336, appears in all nano-PIM compositions as well as the absorption bands at 1738 and 1037 cm^−1^, which correspond to the C=O and C-O-C stretching vibrations in the CTA polymer. The similarity of the positions when compared NP-PIMs with M1 reveal that Aliquat 336 stays entrapped in the polymeric matrix without any chemical change (negligible effect) associated to the presence of the diverse NP. The absorption band at 797 cm^−1^ in Figure 5c) can be assigned to the stretching and deformation vibrations of Si-O-Si siloxane bonds [38]. Other characteristic bands, for example the Fe-O bond at 580 cm^−1^ and 634 cm^−1^ [39,40], and the peak at 690 cm^−1^ assigned to Ti-O stretching bands [41], could not be observed in the corresponding NP-PIMs.

### 3.2. Stability of the NP-PIMs

The stability of PIMs is a critical issue for the application of this membrane type in water treatment. The mass loss of PIMs can be attributed to the finite solubility of the extractant in the adjacent water solution [42,43]. Although PIMs are more stable than their supported liquid membrane counterparts, if relatively soluble extractants are used, such as Aliquat 336, an initial loss of the extractant is observed within the first hours of contact with the adjacent water solution [44]. A mass loss between 10–20% for PIMs containing Aliquat 336 has been reported [5]. In this study, the mass loss of a piece of NP-PIMs was measured after 4 h in contact with SNW. The results are summarized in Table 3. It can be observed that mass loss is around 20% for M1 containing only Aliquat 336. For doped PIMs, similar mass loss is observed. However, improved stability was measured for M11. In particular, M11 containing MWCNTs seems to be the most stable with a measured value of 8.2%. The stability of the PIMs is closely related to the interactions, usually through hydrogen bond formation, between the extractant and the polymer. It seems that the different NPs are not able to produce a modification of the above-mentioned interactions. In the case of MWCNTs, the greater stability could be explained by the ability of MWCNTs to adsorb organic compounds, in particular, Aliquat 336 [45]. To the best of our knowledge, this is the first time that such improved stability related to the presence of MWCNTs has been reported.

In conclusion, the results related to the characterization of the NP-PIMs have shown that the membranes show physical and bulk chemical characteristics similar to the non-doped PIMs. Only in the case of MWCNTs-PIMs was a significant decrease in terms of mass loss observed, making this composition an interesting starting point for the investigation of new and more stable PIMs. The application of the NP-PIMs for the removal of two contaminants of environmental concern, arsenate and phosphate, is described in the following sections for some selected NP-PIM compositions.

### 3.3. NP-PIMs as Sorbents: Removal of Arsenate and Phosphate

PIMs containing Aliquat 336 have been used for arsenate and phosphate transport and detection [34,36,46]. The mechanism for the extraction relies on Aliquat 336 acting as an anion exchanger since As and P are present as anions in water at pH 8.3 (HAsO_4_^2−^ and HPO_4_^2−^, respectively). Therefore, when PIMs are used as sorbents (without a receiving phase), the removal of the target anions is expected to occur via the same anion exchange mechanism.

**Figure 6:**
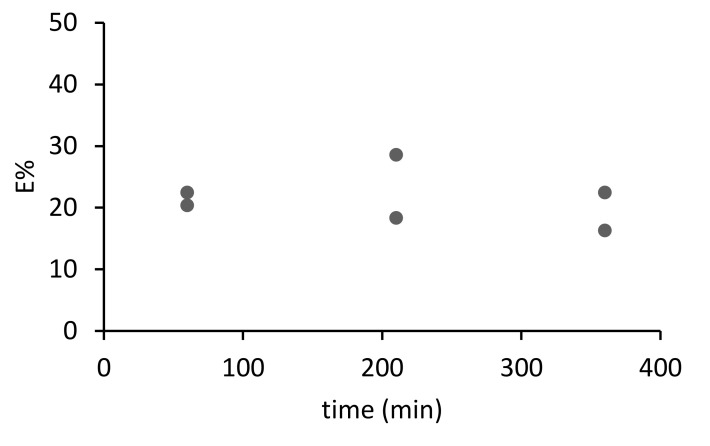
Kinetics of sorption of arsenate (0.5 mg L^−1^ As) with M1 in SNW (*n* = 2)**.**

**Figure 7:**
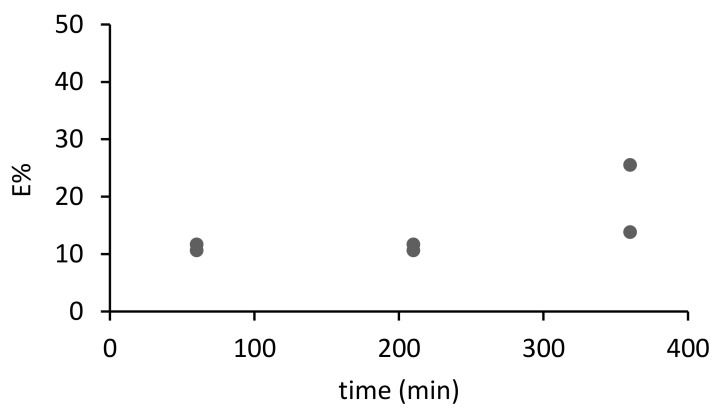
Kinetics of sorption of phosphate (1 mg L^−1^ P) with M1 in SNW (*n* = 2)**.**

Then, the sorption kinetics of arsenate and phosphate were first investigated for M1. The experimental conditions were selected to ensure a molar excess (the ratio mol of extractant: mol of anion) of 25 for phosphate and 125 for arsenate. The percentage of extraction (*E*%) is calculated with Equation (2). As can be observed in Figure 6 and Figure 7, only 20% of As and 10% of P were extracted with the PIM. Even at longer contact times, the amount of the species extracted does not increase. In other sorption systems dealing with Zn (II) extraction, a PIM made of PVC and the extractant D2EHPA [4], the percentage of extraction was much higher at around 90%, when the molar excess of the extractant with respect to the analyte was as low as 10 times. We might conclude that the explanation for the poorer results obtained in the sorption experiments may be related to the high hydration energy of arsenate and phosphate, disfavoring extraction into the PIM phase [47].

To investigate the behavior of PIM doped with the different NPs, a new set of experiments was undertaken with both arsenate and phosphate in SNW at 4 h contact time. The effect of NP in doped PIMs remains an issue to be investigated, but we can speculate that the efficiency of the extraction may be enhanced thanks to the affinity of both arsenate and phosphate to the oxyhydroxide sorbents. Table 4 shows the results for M1 to M10. As can be seen, the PIM doped with 1.7% Fe_3_O_4_ NP (M3) provided 100% extraction for the two analytes and, as such, was the most favorable composition for anion removal. M2, also containing Fe_3_O_4_ NP (0.5%), allows the removal of arsenate but not of phosphate. Finally, other NP-PIMs containing SiO_2_ and TiO_2_ show variable results, which were not completely satisfactory in most cases. Therefore, NP-PIMs based on Aliquat 336 and doped with Fe_3_O_4_ are the best option for the removal of these anions of environmental concern.

The good performance of M3 may be related to the affinity of both arsenate and phosphate for iron oxides as highlighted in the introduction. A blank experiment was performed with a PIM composed of only CTA and Fe_3_O_4_ NP, and no extraction of arsenate was measured. This result suggests that Aliquat 336 helps in the sorption process, promoting the transfer of the anion from the aqueous phase to the organic membrane and contributing to the removal of the anions from the aqueous source solution. The concentration of Fe_3_O_4_ NP in M2 and M3 seems to be an important parameter that is responsible for the selectivity of the NP-PIMs with respect to arsenate and phosphate removal.

### 3.4. Selective Transport of Arsenate and Phosphate

To evaluate not only the removal of arsenate and phosphate, but also their recovery in an aqueous solution, a transport device previously used for arsenate preconcentration was used. In a transport system with PIMs containing Aliquat 336, a high concentration of sodium chloride in the receiving phase promotes the release into the solution (back-extraction) of arsenate or phosphate retained in the PIM through an ion-exchange mechanism where chloride is involved. The recovery of phosphate in the receiving solution affords its preconcentration, a previous step that is necessary, for example, for the valorization of phosphate as a fertilizer. In the case of arsenate, the preconcentration step facilitates the detection of the pollutant in groundwater, as we have demonstrated in a previous study [48].

The experimental conditions have been described in Section 2.5.3 and the transport efficiency for the different NP-PIMs has been calculated using Equation (3). For arsenate (see Table 5), M1, based on Aliquat 336, has demonstrated an efficient transport, of around 90%, after 24 h contact time, in agreement with other results [48]. The addition of the NP promotes changes in the TE% depending on the chemical nature of the NP: NP-PIMs with either SiO_2_, TiO_2_, or MWCNTs also showed quantitative As recovery. However, a dramatic decrease in the transport efficiency was measured for M2, demonstrating the high affinity of the anion for the Fe_3_O_4_ NP as was observed in sorption experiments. Arsenate, being strongly bonded to the iron oxide, cannot be back-extracted into the receiving phase despite the high concentration of chloride used.

The results for phosphate transport were measured for M1, M2, and M10. The average transport efficiency was around 73% for bare PIM. In addition, a similar percentage was obtained for M2 and M10, containing Fe_3_O_4_ NP and TiO_2_ NP, respectively. Therefore, conversely to that was observed for arsenate, phosphate can be preconcentrated in a NaCl solution using NP-PIMs irrespectively of the chemical composition of the NP tested. Unfortunately, MWCNTs-PIMs were not included in the investigation of phosphate transport, which would be interesting from the point of view of evaluating their potential for practical applications. Studies into the recovery of phosphate from the receiving solution through precipitation are currently underway in our laboratory.

## 4. Conclusions

PIMs doped with nanoparticles were effectively prepared and characterized. Flexibility, homogeneity, and the physical appearance were found to be dependent on the type of nanoparticle added. The presence of different nanoparticles does not significantly modify the surface contact angle nor the hydrophilicity of the NP-PIMs, which is basically determined by the presence of Aliquat 336. It was found that the presence of the NP has no effect in mass loss results and only the PIM modified with MWCNTs presents improved stability. The materials were tested for phosphate and arsenate removal in two different experiments: the sorption of the pollutant in the NP-PIM and the transport from the source solution to the receiving solution where the anion is recovered. For sorption experiments, the presence of Fe_3_O_4_ NP improves the percentage of removal from around 20% (PIM without NP) to 80% removal for both phosphate and arsenate. Moreover, the recovery of the species, based on a transport system, is possible for the different types of NP-PIMs, except for arsenate with PIMs modified with Fe_3_O_4_. In conclusion, the presence of SiO_2_ and TiO_2_ has no noticeable effect in the behavior of PIMs made of CTA and Aliquat 336 for arsenate and phosphate removal under the experimental conditions tested. The improved stability of MWCNTs-PIMs, and the high extraction capacity of Fe_3_O_4_-PIMs, are important findings for future work.

## Figures and Tables

**Table 1:** Composition (% *w*/*w*) of the NP-PIMs prepared for the present study.

	CTA	Aliquat 336	SiO_2_	TiO_2_	Fe_3_O_4_	MWCNTs
M1	50.0	50.0				
M2	52.0	47.0			0.5	
M3	51.2	47.1			1.7	
M4	52	47	1			
M5	49.5	45.7	4.8			
M6	47.3	43.5	9.2			
M8	51.3	47.7		1		
M9	49	46.1		4.9		
M10	47.9	43		9		
M11	44.4	41				14.6

**Table 2:** Results of contact angle measurement.

NP-PIM	Contact Angle (Average ± sd) (°)
M1 (CTA-Aliquat 336)	17 ± 3
M2 (Fe_3_O_4_)	23 ± 2
M3 (Fe_3_O_4_)	24 ± 2
M4 (SiO_2_)	25± 1
M5 (SiO_2_)	15 ± 2
M6 (SiO_2_)	22 ± 6
M8 (TiO_2_)	21 ± 2
M9 (TiO_2_)	23 ± 2
M10 (TiO_2_)	20 ± 1
M11 (MWCNTs)	17 ± 4

**Table 3:** Mass loss data for the NP-PIMs after 4 h in synthetic natural water (SNW). Results for two replicates are presented in brackets.

NP-PIM	Mass Loss
M1 (CTA-Aliquat 336)	(19.3, 21.2)
M2 (Fe_3_O_4_)	(19.4, 20.2)
M3 (Fe_3_O_4_)	(14.8, 12.6)
M4 (SiO_2_)	18.3
M5 (SiO_2_)	14.3
M6 (SiO_2_)	(16.8, 17.2)
M8 (TiO_2_)	(16.0, 17.4)
M9 (TiO_2_)	(17.8, 19.8)
M10 (TiO_2_)	17.2
M11 (MWCNTs)	(7.5, 8.9)

**Table 4:** Sorption efficiency data for the NP-PIMs after 4 h in SNW. Results for two replicates are presented in brackets.

NP-PIM	E% for Arsenate	E% for Phosphate
M1 (CTA-Aliquat 336)	(22, 18)	(12, 10)
M2 (Fe_3_O_4_)	(83, 79)	(11, 13)
M3 (Fe_3_O_4_)	100	97
M4 (SiO_2_)	41	0
M5 (SiO_2_)	58	0
M6 (SiO_2_)	31	30
M8 (TiO_2_)	(56, 54)	(0, 5)
M9 (TiO_2_)	41	31
M10 (TiO_2_)	n.d.	5

n.d. not determined.

**Table 5:** Transport efficiency for arsenate (0.1 mg L^−1^ As in SNW), after 24 h contact time. Receiving solution 2 M NaCl.

NP-PIM	TE% for Arsenate
M1 (CTA-Aliquat 336)	(92, 88)
M2 (Fe_3_O_4_)	36
M3 (Fe_3_O_4_)	n.d.
M4 (SiO_2_)	n.d.
M5 (SiO_2_)	87
M6 (SiO_2_)	n.d.
M8 (TiO_2_)	92
M9 (TiO_2_)	100
M10 (TiO_2_)	n.d.
M11 (MWCNTs)	100

n.d. not determined.

## Data Availability

The data presented in this study are available on request from the corresponding author.
